# Longitudinal trajectories of amyloid deposition, cortical thickness, and tau in Down syndrome: A deep-phenotyping case report

**DOI:** 10.1016/j.dadm.2019.04.006

**Published:** 2019-11-25

**Authors:** Elijah Mak, Anastasia Bickerton, Concepcion Padilla, Madeleine J. Walpert, Tiina Annus, Liam R. Wilson, Young T. Hong, Tim D. Fryer, Jonathan P. Coles, Franklin I. Aigbirhio, Bradley T. Christian, Benjamin L. Handen, William E. Klunk, David K. Menon, Peter J. Nestor, Shahid H. Zaman, Anthony J. Holland

**Affiliations:** a Department of Psychiatry, University of Cambridge, Cambridge, UK; b Wolfson Brain Imaging Centre, Department of Clinical Neurosciences, Cambridge, UK; c Division of Anaesthesia, University of Cambridge, Cambridge, UK; d Waisman Center, University of Wisconsin-Madison, Madison, WI, USA; e Department of Psychiatry, University of Pittsburgh, Pittsburgh, PA, USA; f Queensland Brain Institute, University of Queensland, Brisbane, Australia

## Abstract

**Introduction:**

Comorbid Alzheimer disease pathologies are frequently found in people with Down syndrome (DS). We report a deep phenotyping study undertaken over 7 years in a participant with DS who was nondemented at baseline but developed dementia after 5 years.

**Methods:**

Throughout the course of the study, the participant was seen 4 times (2010, 2013, 2015, and 2017). Multimodal neuroimaging, including three serial scans of [^11^C]-PiB-PET, four structural magnetic resonance imagings, as well as a [^18^F]-AV1451 scan, was interpreted alongside detailed neuropsychological assessments over the study period.

**Results:**

Amyloid beta accumulation preceded the onset of dementia and cognitive decline, which in turn corresponded to the predominant deposition of tau in temporoparietal cortices.

**Discussion:**

Until now, data on the longitudinal trajectories of amyloid accumulation, tau pathology, and brain atrophy over multiple time points remain scarce in DS. This case report highlights the potential for deep phenotyping imaging to elucidate the substrates of cognitive decline in DS, although further longitudinal studies are necessary to clarify the relative contributions of both amyloid and tau.

## 1 Introduction

Down syndrome (DS) is the most common neurodevelopmental disorder caused by the presence of trisomy 21 (1:800 live births). The extra copy of amyloid precursor protein (*APP*) gene on chromosome 21 is associated with a 4- to 5-fold overexpression of amyloid precursor protein that results in increased cerebral accumulation of its proteolytic product of β-amyloid (Aβ) [1]. Consequently, Alzheimer's disease (AD) pathologies (i.e., Aβ and neurofibrillary tau tangles) and comorbid dementia are highly prevalent in people with DS by the fifth decade [2]. Clarifying the neuropathological substrates that underpin cognitive decline may help identify suitable therapeutic interventions and optimization of clinical trials. Until now, data on the longitudinal trajectories of amyloid accumulation, tau pathology, and brain atrophy over multiple timepoints remain scarce. We report a deep-phenotyping case study undertaken over 7 years (4 timepoints) in a participant with DS who was nondemented at baseline but who developed clinical dementia after 5 years. Multimodal neuroimaging modalities, including three serial scans of [^11^C]-PiB-PET, four structural magnetic resonance imaging (MRI) scans, and a [^18^F]-AV1451 scan, were interpreted alongside in-depth cognitive assessments over the study period.

## 2 Methods

### 2.1 Clinical assessment and cognitive profile

The participant enrolled into the study when he was 48 years old in 2010. His cognitive function was assessed using the CAMCOG, a validated tool for assessing cognitive decline in DS. He was also assessed using the Cambridge Examination for Mental Disorders of Older people with Down's Syndrome and Others with Intellectual Disabilities informant interview, a neuropsychological assessment battery designed for diagnosing dementia in accordance with the International Classification of Diseases-10 criteria for dementia in DS [3]. The studies that he was part of were all approved by the National Research Ethics Committee of East of England and the Administration of Radioactive Substances Advisory Committee.

### 2.2 Multimodal imaging

#### 2.2.1 Structural MRI

Across timepoints 1–4, the T1-MPRAGE data were processed using the longitudinal pipeline of FreeSurfer to obtain measurements of cortical thickness measurements in 34 region-of-interest per hemisphere, based on the Desikan-Killiany parcellation scheme [4]. The technical procedures for surface reconstruction and quantification of cortical thickness have been described previously. Briefly, the process involved automated nonuniformity bias correction, skull stripping, segmentation of the white matter, and the boundary between the white and gray matter. The gray/white boundary served as a starting point for a deformable surface algorithm to compute the gray/white and pial surfaces, from which cortical thickness is calculated as the closest distance from the gray/white matter boundary to the pial surface at each vertex on the tessellated surface. In addition, PetSurfer was used to segment additional regions, such as the cerebrospinal fluid, pons, skull, and air cavities to facilitate partial volume correction of PET data. Each PET datum was registered to the resulting template, and partial volume correction was performed in PetSurfer, consistent with our previous methodology [5].

#### 2.2.2 [^11^C]-PiB and [^18^F]-AV1451 imaging

[^11^C]-PiB data were acquired in three-dimensional mode on a GE Advance scanner. Before [^11^C]-PiB injection, a 15-minute transmission scan using rotating ^68^Ge rod sources was acquired to correct for photon attenuation. [^11^C]-PiB was produced with high radiochemical purity (>95%) and specific activity (>150 GBq/umol). [^11^C]-PiB was injected as a bolus (median = 545 MBq, interquartile range = 465-576 MBq) through an antecubital venous catheter, and data were acquired for 90 min after injection in 58 frames (18 × 5, 6 × 15 seconds, 10 × 30 seconds, 7 × 1 minute, 4 × 2.5 minutes, and 13 × 5 minutes). For each frame, sonogram data were reconstructed using the PROMIS three-dimensional filtered back-projection algorithm into a 128 × 128 x 35 image array with a voxel size of 2.34 × 2.34 × 4.25 mm [3]. The dynamic PET images were realigned with statistical parametric mapping and averaged. The resultant mean images were coregistered to FreeSurfer-processed T1 data for quantification in FreeSurfer region-of-interest using PetSurfer. Tau deposition was quantified *in vivo* using [^18^F]-AV-1451 on a GE SIGNA PET/MR scanner. Standardized uptake value ratio for [^18^F]-AV-1451 was calculated for data 75–105 minutes after injection (185 MBq) with the cerebellum as the reference region. Images (6 × 5 minutes; 2.0 × 2.0 × 2.8 mm^3^) were reconstructed using OSEM (16 subsets, 6 iterations; no smoothing), with a low-dose CT scan acquired on a GE Discovery 690 PET/CT used for attenuation correction of the head. Finally, [^18^F]-AV-1451 standardized uptake ratio was calculated with the cerebellum as the reference region.

## 3 Result

**Fig. 1:**
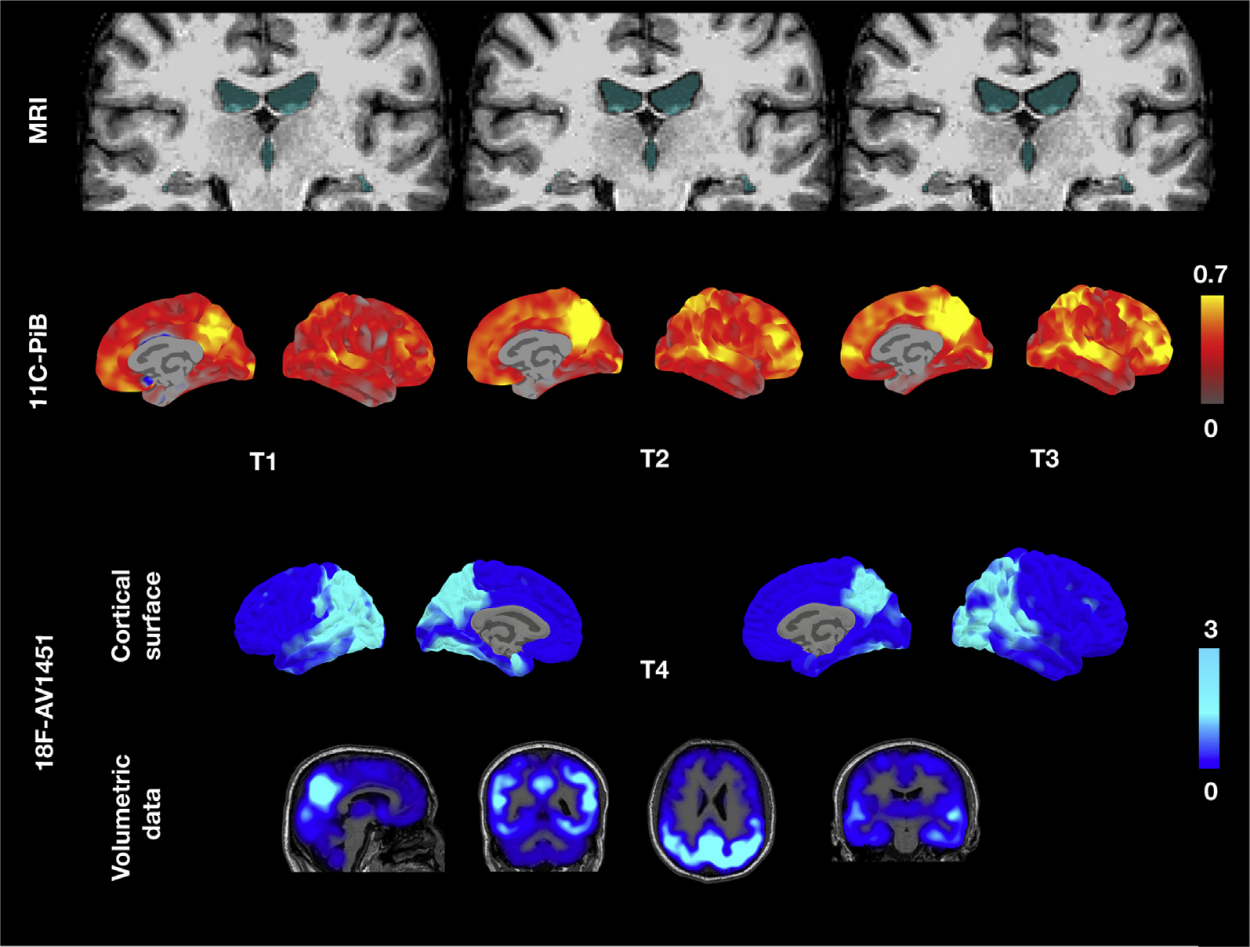
Trajectory of structural atrophy, Aβ accumulation over 3 timepoints and tau deposition at timepoint 4. Top row: Across the timepoints, structural MRI revealed progressive ventricular dilatation and subtle hippocampal atrophy with respect to baseline (blue voxels). Middle row: Peak regions of amyloid accumulation were distributed in posterior regions, such as the precuneus. Over the course of study, the spatial extent of amyloid increased to encompass temporal regions. Bottom row: At T4, [^18^F]-AV1451 scan showed a stereotypical pattern of AD tau distribution that involved the bilateral precuneus and temporoparietal cortices. Abbreviations: Aβ, amyloid β; AD, Alzheimer's disease; MRI, magnetic resonance imaging.

**Fig. 2:**
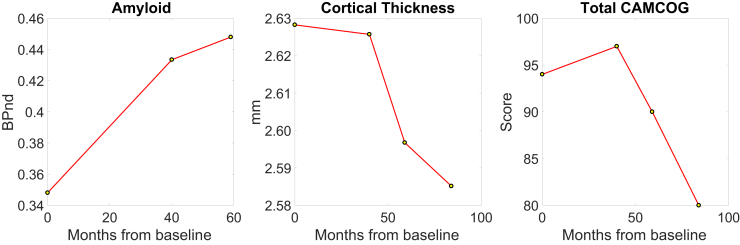
Contrasting longitudinal trajectories associated with Aβ accumulation and cortical thinning across the cortex. The sharp increase of Aβ at T2 relative to T1 was followed by a plateau at T3, whereas cortical thickness continued to decrease across all timepoints. Abbreviation: Aβ, amyloid β.

**Fig. 3:**
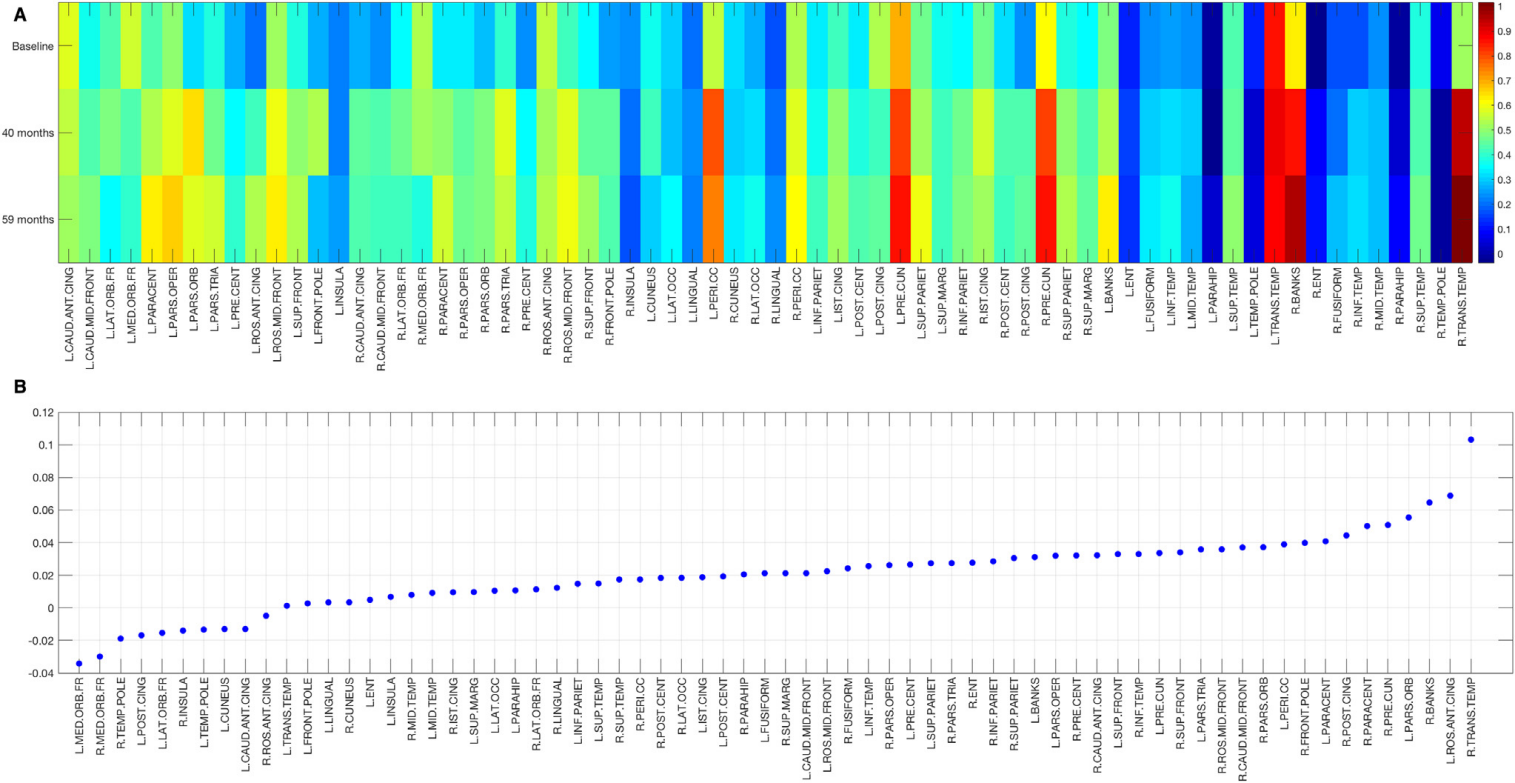
Regional distribution of Aβ accumulation from baseline to 59 months. A: Heatmap of [11C]-PiB BP_ND_ across the cortex. B: Annualized rate of Aβ accumulation in cortical regions. Abbreviation: Aβ, amyloid β.

Throughout the course of the study, the participant was seen 4 times (2010, 2013, 2015, and 2017). The participant was enrolled into the study with a diagnosis of mild cognitive impairment. Clinically, he had a history of depressive symptoms without any behavioral problems. On his initial assessment (2010), he scored 94/109 on the CAMCOG (Table 1). Baseline [^11^C]-PiB scans revealed elevated Aβ accumulation, most pronounced in the bilateral precuneus and striatum. Structural MRI revealed no signs of severe atrophy with a medial temporal lobe atrophy score of 1 (Fig. 1). At the third visit in 2015, the patient was confirmed to have clinical dementia. During this period, behavioral changes emerged, such as a lack of enthusiasm, increased tendencies for verbal repetition, and stubbornness. Memory impairments were also noted, which included an increased difficulty with remembering the locations of items and content of conversations, and the time of day. Language was also affected, evidenced by an increased difficulty with keeping up with ordinary conversation and word-finding difficulties alongside an overall slowing in thinking. The onset of the dementia was preceded by a sharp Aβ accumulation, from 0.35 [^11^C]-PiB non-displaceable binding potential (BP_ND_) at baseline (2010) to 0.45 [^11^C]-PiB BP_ND_ at T2 (2013), before Aβ reached a plateau at T3 (2015; [^11^C]-PiB BP_ND_ = 0.45; Fig. 2). Relative to the baseline [^11^C]-PiB scan, increased Aβ burden at T2 and T3 was observed in frontoparietal cortices with relative sparing of the temporal lobe (Fig. 3A). Of note, the fastest rate of annualized Aβ accumulation was estimated to be 0.1 BP_ND_ (Fig. 3B). Similarly, structural MRI revealed progressive atrophy and ventricular dilatation, although it was most pronounced only at T3 (Fig. 1). Visual inspection of the [^18^F]-AV1451 at T4 revealed two main observations: (i) a stereotypical pattern of elevated tau deposition, characterized by predominant binding in the bilateral precuneus and temporoparietal cortices (Fig. 1) and (ii) in contrast to the often-reported striatal pattern of [^11^C]-PiB signal in DS, this region was relatively spared of [^18^F]-AV1451 binding (Fig. 1).

**Table 1:** Raw scores achieved in different functional areas evaluated in the CAMCOG across 7 years: Scores collected over 4 timepoints

Cognitive variables	T1	T2	T3	TX∗	T4	Max score
Orientation total	10	10	12	12	12	12
Comprehension	7	9	6	7	2	9
Expression	16	18	17	18	14	18
Language total	23	27	23	25	16	27
New learning	17	15	15	18	17	21
Remote	4	4	4	4	0	4
Recent	4	4	4	2	2	4
Memory total	25	23	23	24	19	29
Attention total	8	9	7	8	8	9
Drawing	6	7	6	5	6	8
Actions to command	10	9	9	8	8	10
Praxis total	16	16	15	13	14	18
Abstract thinking total	4	4	2	2	3	6
Perception total	8	8	8	8	8	8
CAMCOG total	94	97	90	92	80	109

∗ TX = No corresponding brain scan data. Maximum score is shown in the last column for each area.

## 4 Discussion

In this deep-phenotyping case study of an adult with DS, we reported a sharp increase in Aβ burden that preceded the onset of clinical dementia and pronounced decline in memory and language functions, which, in turn, mapped onto the topography of tau deposition within the temporoparietal cortex. To the best of our knowledge, this is the first *in vivo* demonstration of progressive Aβ accumulation during the process of dementia conversion from mild cognitive impairment. The close mapping between AD pathologies and clinical decline further demonstrates the potential utility for deep-phenotyping studies to elucidate the temporal relationships between *in vivo* pathological AD markers in DS.

Of clinical interest, the patient's transition from a prodromal phase of dementia to dementia was preceded by a sharp increase in Aβ burden (2010–2013). As shown in Fig. 1A, some regions showed low binding that stayed low (e.g., entorhinal cortex, fusiform gyrus, parahippocampal cortex), whereas others were already close to plateau at baseline and therefore did change further, but some regions were captured during the transition, mostly encompassing frontal and parietal cortical regions. These data showed that the maximum rate of change was 0.1 [^11^C]-PiB BP_ND_ per year. One caveat to this estimate, however, is that amyloid accumulation in DS may occur at a more aggressive rate than in the general population.

In keeping with hypothetical models of sporadic AD [6], our data suggest that the dynamic phase of Aβ deposition in DS occurs during the prodromal phase and little change in Aβ deposition is expected after the onset of clinical dementia. This follows that earlier interventions, ideally in the prodromal phase of DS, are urgently needed to gain the maximum therapeutic potential of drugs that target the Aβ pathways. We also observed a contrasting pattern of trajectories between Aβ accumulation and brain atrophy in this patient with DS, where progressive increase in Aβ paralleled with cortical thinning over the course of the study. This coupling between increased amyloid and brain atrophy is broadly consistent with our previous work on a larger cohort of DS. In the absence of further Aβ accumulation at T3, we speculate about the neuropathological substrates underpinning the worsening atrophy and cognitive decline between T3 and T2, with hyperphosphorylated tau being the prime suspect. Indeed, the patient with DS showed elevated [^18^F]-AV1451 binding in the temporoparietal cortices, where we have recently demonstrated a tight coupling between tau pathology and atrophy in AD [5]. The regional distribution of tau in our patient with DS also resembled the stereotypical distribution of tau hyperphosphorylation associated with AD (see Hall and Mak et al., [7], for a systematic review of tau PET imaging). Further studies, including our ongoing work in the Neurodegeneration in Aging Down Syndrome study with tau-tracer [^18^F]-AV1451 and [^11^C]-PiB imaging, will help disentangle the differential contributions of Aβ and tau toward brain atrophy and disease progression.

In summary, this brief report demonstrates the utility for deep-phenotyping designs to improve our understanding of the disease course and highlight the potential for multimodal imaging to elucidate the interactions among AD neuropathologies in driving cognitive dysfunction. Further replication of these findings would inform future studies and therapeutic trials for which the DS population is a prime candidate.

**Research in Context**

1. Systematic review: Adults with Down syndrome (DS) often have coexisting Alzheimer's disease (AD) neuropathologies that are considered to be consequent on the triplication of chromosome 21. Although there has been significant progress in our understanding of the role of amyloid β in the disease course of DS, longitudinal evidence capturing the process of amyloid accumulation and how it relates to worsening cognitive impairment is still unclear. There are currently no longitudinal studies or case reports relating *in vivo* amyloid burden or tau deposition to cognitive outcomes.
2. Interpretation: Our case study showed—for the first time—that a sharp increase in amyloid β preceded the onset of clinical dementia. In addition, the [^18^F]-AV1451 PET scan revealed a stereotypical AD-like distribution of tau deposition, particularly in the temporoparietal cortices. These observations are consistent with biomarker models in AD and highlight the potential for deep-phenotyping imaging to disentangle the contributions of AD neuropathologies in DS.
3. Future directions: Further longitudinal studies are necessary to clarify the extent to which amyloid β and tau deposition contribute to subsequent cognitive decline in DS.
